# CD28^−^ Cells Are Increased in Early Rheumatoid Arthritis and Are Linked With Cytomegalovirus Status

**DOI:** 10.3389/fmed.2020.00129

**Published:** 2020-05-05

**Authors:** Charlotte Thompson, Ruth Davies, Anwen Williams, Gareth Jones, Ernest H. S. Choy

**Affiliations:** ^1^CREATE Centre, Section of Rheumatology, Division of Infection and Immunity, Cardiff University School of Medicine, Cardiff, United Kingdom; ^2^Department of Clinical and Experimental Medicine, Brighton and Sussex Medical School, University of Sussex, Brighton, United Kingdom; ^3^School of Cellular and Molecular Medicine, Biomedical Sciences Building, University of Bristol, Bristol, United Kingdom

**Keywords:** CMV, Rheumatoid Arthritis, DAS28, CRP, RF, ACPA, CD28^−^, T cells

## Abstract

**Objective:** CD3^+^CD8^+^CD28^−^ cells are higher in Rheumatoid Arthritis (RA). The aim of this study was to assess CD3^+^CD8^+^CD28^−^ cells in patients with early RA and assess the effects of cytomegalovirus (CMV) seropositivity.

**Method:** In this prospective observation study, 50 RA patients were recruited from Cardiff University Hospital of Wales (UHW) rheumatology outpatient, 25 patients with early disease (disease duration 0–6 months) and 25 patients with established disease (>2 years). These were compared with 25 healthy controls. Clinical and serological markers of inflammation were noted, and peripheral blood mononuclear cells were analyzed using flow cytometry.

**Results:** The percentage of the CD8^+^CD28^−^ T cells was increased in RA patients and was associated with disease duration. The percentage of CD8^+^CD28^−^ T cells was increased in CMV positive early and established RA grouped and early RA patients in comparison to CMV negative patients (*p* < 0.05). There is a weak but statistically significant correlation between the percentage of CD3^+^CD8^+^CD28^−^ cells and CRP in CMV positive RA patients (*r* = 0.227, *p* < 0.05).

**Conclusion:** The percentage of CD8^+^CD28^−^ T cells is higher in RA patients and correlates with disease duration, highlighting a potential role early in the disease process. These cells were also higher in CMV positive early RA patients which may suggest a role of CMV in disease development.

## Key Messages

- CD3^+^CD8^+^CD28^−^ cells are increased in RA and correlated with disease duration.
- CD3^+^CD8^+^CD28^−^ cells are higher in CMV positive patients.
- CD3^+^CD8^+^CD28^−^ cells are weakly associated with CRP.

## Introduction

CD8^+^ T cells are important in controlling viral infection. Acute viral infection promotes the expansion of CD8^+^ cells, which express CD45Ro, CD38 and HLA-DR, CD28, and CD27. CD28 is a key co-stimulatory molecule expressed on naïve CD4^+^ and CD8^+^ T cells. Activation of the T cell receptor with antigen presenting cells (APCs) via MHC-I-bound peptide antigen has low affinity and is therefore insufficient to induce full activation and survival of T cells. The co-stimulatory signal from CD28 cell surface receptor which interacts with CD86 or CD80 on antigen presenting cells, is needed to prolong T cell responses (1).

Alongside CD8^+^ T cells CD4^+^ T cells also play a key role in the defense against CMV infection. CMV infection increases the number of CD4^+^CD28^−^ T cells (2). CMV specific CD4^+^ T cells precede the appearance of CMV specific CD8^+^ T cells during primary infection, but are delayed in symptomatic patients (3, 4).

Several studies have reported significant differences between RA patients and healthy controls with respect to the frequency of CD4^+^CD28^−^ T cells (5–7). However, few have accounted for CMV seropositivity. RA patients have significant expansions of the CD4^+^CD28^−^ T cells in CMV positive compared to CMV negative and this is associated with more severe joint destruction (8).

Persistent T cell stimulation and proliferation down-regulate CD28 expression and causes shortening of telomeres in these cells (9). The loss of CD28 on CD8 cells has also been considered a marker of immunosenescence and highly differentiated or late differentiated cells (10). High TNF concentrations, as in the case with RA, also abrogate CD28 transcription (11). The loss of CD28 is combined with increased expression of CD57 (12). The proportion of CD8^+^CD57^+^ and CD8^+^CD28^−^T cells were significantly higher in RA patients compared with age-matched controls (13). However, their exact role in RA in unknown. Some evidence suggest they may be pro-inflammatory as they express TNF and IFN-γ (14). We have shown CD8^+^CD28^−^ T cells from healthy individuals suppressed autologous mixed lymphocyte proliferation although this was impaired in patients with RA (15). This dysfunction was partially reversed by TNF inhibitor therapy. Furthermore, the immunosuppressive form of IFN-γ correlates with CD8^+^CD28^−^ T cells, so might be considered as CD8^+^ regulatory T cells (Treg) (15, 16).

Cytomegalovirus (CMV), can shape the human memory T cell compartment by generating terminally differentiated T cells, which are characterized by the loss of CD27 and CD28, shortened telomeres (17), and by the expression of inhibitory natural killer (NK) cell receptors. CD8^+^ T cells restrict CMV replication but do not eliminate or stop transmission. Repeated rounds of antigen-driven proliferation by CMV continue to cause the clonal expansion of CMV-specific CD8^+^ T cells, which accumulate with age. Chronic CMV infection expands a population of CD8^+^ T cells that can efficiently control latent infection, while certain effector function are reduced to prevent harm due to collateral autoreactivity (18). Latent CMV infection and RA share several phenotypical features in the T cell compartment. CD8^+^CD28^−^ T cells are expanded in healthy individuals chronically infected with CMV, and even more so in CMV infected RA patients (19). CMV positivity has been associated with more severe joint destruction in RA (8) and CMV IgG positivity is lower in controlled RA compared to active RA (20).

The aim of this paper is to establish whether CD8^+^CD28^−^ T cells are raised in Early as well as Established RA, and furthermore to examine the effect of prior CMV exposure on this subtype of cells.

## Method

### Patients

The study was approved by the South East Wales Research Ethics Committee, Panel B in 2011 (REC reference: 11/WA/0326). Cardiff University was responsible for the governance of the study with reference number 11/CMC/5299. The Cardiff and Vale University Health Board Research & Development Office approved the proposal in 2013.

In this prospective observation study, 50 RA patients (25 early and 25 established disease) were recruited from University Hospital of Wales (UHW) rheumatology outpatient clinic in Cardiff. Twenty-five healthy controls were recruited from Cardiff University.

Inclusion criteria included the following: age ≥18 years; ACR/EULAR 2010 criteria of RA diagnosis (21); duration of persistent symptoms in the Early RA group of 4 weeks to 6 months or >2 years since diagnosis for the established RA group.

Exclusion criteria include the following: other autoimmune/inflammatory rheumatic disease; heart disease classified as New York Heart Foundation Functional class IV (ACR classification); treatment with intravenous gamma globulin, plasmapheresis, or Prosorba^TM^ column within the last 6 months.

### Clinical Assessments

Clinical assessments included age, gender, RA disease duration, Disease Activity Score 28 (DAS28) score and blood was taken to asses Rheumatoid Factor (RF), Anti-citrullinated Peptide Antigen (ACPA), Erythrocyte Sedimentation Rate (ESR), C-Reactive Protein (CRP), and CMV status (IgG positive or negative).

### Immunofluorescence and Flow Cytometry

Peripheral blood mononuclear cells (PBMCs) were collected from subjects into two 4 ml BD Vacutainers^TM^ then centrifuged and purified. T cell staining with fluorescently conjugated antibodies using anti-CD3 (APC-Cy7, BD Biosciences), anti-CD8 (Pe-Vio-770, Miltenyi Biotec Ltd.), anti-CD28 (eFluor 450, eBioscience) were prepared for flow cytometric analysis of cells. Cells were acquired using a CyAn ADP (Beckman Coulter) flow cytometer and analyzed using Summit software (software version 4.3; Beckman Coulter).

### Statistics

Statistical analysis was performed using GraphPad Prism v5 software. Pairwise comparisons were determined using the Mann Whitney *U*-Test. Comparison of multiple groups was assessed using ANOVA with Holm-Sidak correction for multiple comparison. For non-parametric data, Kruskal-Wallis and *post-hoc* Dunn's tests were used. *P* < 0.05 was considered significant. The Spearman rank correlation was used to analyse statistical associations.

## Results

Demographic data and clinical characteristics are shown in Table 1. There were 25 patients with Early RA, 25 patients with Established RA and 25 healthy controls. The mean age of the patient groups was similar (56 in Early RA and 62 in Est RA). The mean age of the healthy controls was 41. Disease activity was higher in the Est RA group (DAS28: 5.4 and 4.07, respectively). The proportion of female subjects in each group was similar (between 64 and 68%). Twenty-five Early RA patients were tested for CMV (52% positive), 12 of the Est RA were tested (42% positive).

Table 1Demographics of health controls, early, and established RA patients recruited **(A)** and demographics of the patients tested for CMV positivity at baseline **(B)**.

**Table:** 

**(A)**			
	**Healthy controls (*n* = 25)**	**Early RA (*n* = 25)**	**Established RA (*n* = 25)**
Age	41 ± 12	56 ± 12	62 ± 12
Disease duration	N/A	4 ± 1.2 months	10 ± 7.6 years
RF positive (%)	N/A	17 (68)	14 (56)
ACPA positive (%)	N/A	19 (79)	15 (63)
DAS28	N/A	4.07 ± 1.3	5.4 ± 1.9
Female (%)	17 (68)	16 (64)	17 (68)

**Table:** 

**(B)**						
	**Early RA**		**Established RA**		**Pooled RA**	
	**CMV negative**	**CMV positive**	**CMV negative**	**CMV positive**	**CMV negative**	**CMV positive**
Number of patients	12	13	7	5	19	18
Duration disease	3.7 ± 1.2 months	4.1 ± 1.5 months	8.1 ± 9.0 years	11.8 ± 5.9 years	N/A	N/A
Age	52 ± 15	61 ± 8	65 ± 10	66 ± 11	56 ± 14	56 ± 9
RF positive (%)	9 (75)	7 (54)	3 (43)	4 (80)	12 (63)	11 (61)
ACPA positive (%)	10 (83)	9 (69)	3 (43)	3 (75)	13 (68)	12 (67)
Mean DAS28	4.0 ± 1.1	4.2 ± 1.6	5.3 ± 1.0	4.8 ± 2.3	4.4 ± 1.2	4.3 ± 1.8
Female %	58 (7)	77 (10)	71 (5)	80 (4)	68 (13)	78 (14)

*Percentages are shown with number of subjects in brackets. Mean average is presented. Age and DAS28 are shown with standard deviation*.

### CD8^+^CD28^−^ T Cells in Early and Established RA

**Figure 1:**
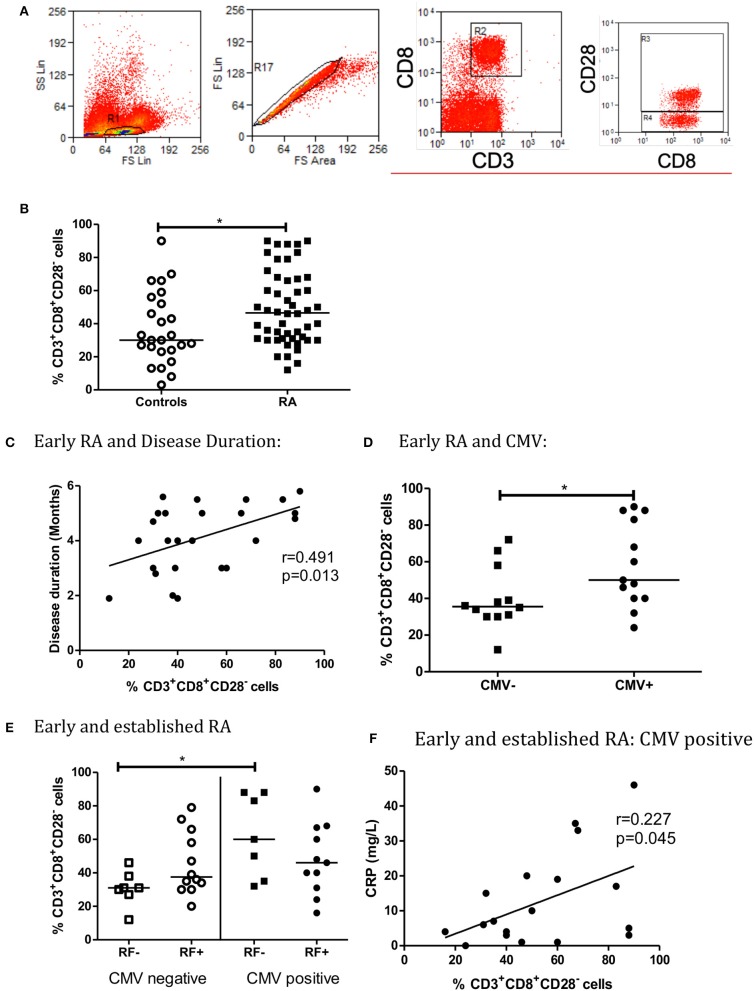
The percentage of CD3^+^CD8^+^CD28^−^ T Cells is higher in early and established RA grouped **(B)**. Flow cytometry gating strategy for lymphocytes, single cells and CD3^+^CD8^+^ and CD3^+^CD8^+^CD28^**−**^ **(A)**. There is low level correlation between the percentage of CD3^+^CD8^+^CD28^−^ T cells and Disease Duration in Early RA patients **(C)**. Peripheral blood CD3^+^CD8^+^CD28^−^ T cells are also increased in CMV positive early RA patients (**C**, *n* = 25, CMV positive = 13, CMV negative = 12). When RA patients are Rheumatoid Factor (RF) negative, the percentage of CD3^+^CD8^+^CD28^−^ cells is higher in CMV positive patients (**E**, *n* = 37). There is a weak statistically significant correlation between the percentage of CD3^+^CD8^+^CD28^−^ cells and C reactive protein (CRP) in CMV positive early and established RA grouped patients **(F)**. Percentage of CD3^+^CD8^+^CD28^−^ T Cells are shown with median, *p < 0.05 by Mann-Whitney *U*-Test **(B,D)** or One-way ANOVA Test with Holm-Sidak analysis for multiple comparisons **(E)**. Controls (*n* = 24), early and established RA grouped (*n* = 50), Early RA (*n* = 25), Established RA (*n* = 25). Correlation was determined using non-parametric Spearman's rank analysis, *p < 0.05. Early and established grouped RA (**E,F**, *n* = 37), Early RA (*n* = 25), Est RA (*n* = 12), CMV positive (*n* = 18).

Percentage of CD8^+^CD28^−^ T cells in Early and Established RA was statistically significantly higher (%) than Controls (*p* = 0.048, Figure 1B).

In early RA patients, percentage of CD8^+^CD28^−^ T cells correlated with disease duration (*r* = 0.491, *p* = 0.013, Figure 1C). Percentage of CD8^+^CD28^−^ T cells was increased in CMV positive early RA patients in comparison to CMV negative early RA patients (Figure 1D). In contrast, the percentage of CD8^+^CD28^−^ T cells did not correlate with disease duration in established RA (*r* = 0.164, *p* = 0.433) data not shown. There was no correlation with measurement of disease activity by disease activity score 28 (DAS28), which includes a tender and swollen joint count, ESR or CRP and pain score (early RA: *r* = 0.003, *p* = 0.812, established RA: *r* = 0.020, *p* = 0.524).

For RF negative patients, the percentage of CD8^+^CD28^−^ T cells was higher in CMV positive grouped early and established RA patients, than CMV negative (*p* < 0.05, Figure 1E). The association between RF and CD4^+^CD28^−^ T cells in RA and control patients has previously been investigated, and no statistical difference was observed (*p* = 0.062) in our study (22). Similarly, CD8^+^CD28^−^ T cells percentage was not associated with ACPA seropositivity (data not shown). Both RF and ACPA are autoantibodies that signify a poorer prognosis in RA.

There is a weak but statistically significant correlation between the percentage of CD8^+^CD28^−^ T cells and CRP in CMV positive early and established RA patients (*r* = 0.227, *p* = 0.045, Figure 1F). There was no correlation between CD3^+^CD8^+^CD28^−^ T cells and ESR (CMV positive early and established RA patients: *r* = 0.439, *p* = 0.069) data not shown. CRP has not previously been found to be associated with CD8^+^CD28^−^ or CD4^+^CD28^−^ T cell accumulation in the context of CMV seropositivity (23).

## Discussion

CD8^+^CD28^−^ T cells have been shown to be raised in RA. In this study, we found that increase in CD8^+^CD28^−^ T cells occurred early and correlate with disease duration suggesting they have a possible role early in the disease process. The expansion of this cell subset could be contributing to immune disturbance in RA. Alternatively, these cells could reflect a bystander population that reflects an active autoimmune process in early disease. The number of studies on the role of CD8+ T cells in the pathogenesis of RA is scant However, increased frequency of CD8+ T cells are found in the lymph node and peripheral blood of patients with RA suggest they may be important in early disease development (24).

Similar to the previous study by Ceeraz et al. (15), we also found no significant correlation with the disease activity score and CD8^+^CD28^−^ T cells in established RA (6). However, in this study, we found statistically significant correlation between CRP and CD8^+^CD28^−^ T cells in early RA suggesting their function may change during chronic disease.

CMV status was associated with the percentage of the CD8^+^CD28^−^ T cells which was increased in CMV positive early RA patients in comparison to CMV negative early RA patients. CD8^+^CD28^−^ T cells have been found to be significantly increased in CMV positive compared to CMV negative in other autoimmune diseases (25). Our findings that percentage of the CD8^+^CD28^−^ T cells was increased in CMV positive early RA patients in comparison to CMV negative early RA patients was in keeping with this. Although in RA patients the number of EBV-specific CD8^+^ T-cells correlates positively with the viral load, the CD8^+^ T-cell responses to CMV antigens do not (26).

Increased in CD8^+^CD28^−^ cells could be due to prior CMV infection because CMV infection is known to increase the absolute number of CD8^+^CD28^−^lymphocytes (27). Indeed, this was the case in this study. Latent CMV infection may prime memory T cells response so that when RA develops, this may lead to more severe disease (10).

A limitation of this study was that the number of Established RA patients that were tested for CMV was low (12/25). Therefore, subdivision into Established RA would not have sufficient statistical power for sub-analysis into this group. Increasing the sample size, particularly the Established RA group, would increase the power of the study and allow for further sub-group analysis.

In conclusion, the percentage of CD8^+^CD28^−^ T cells is higher in early RA and further increased in CMV positive early RA patients. In RF negative RA patients, the percentage of CD8^+^CD28^−^ T cells is higher in CMV positive patients and there is weak positive correlation of CD8^+^CD28^−^ T cells with CRP.

## Conclusions

The percentage of CD8^+^CD28^−^ T cells is higher in RA patients and correlates with disease duration in early RA patients, highlighting a potential role early in the disease process. These cells were also higher in CMV positive early RA patients which may suggest a role of CMV in disease development.

## Data Availability Statement

The datasets generated for this study are available on request to the corresponding author.

## Ethics Statement

This study including human participants was approved by the South East Wales Research Ethics Committee, Panel B in 2011 (REC reference: 11/WA/0326). Cardiff University was responsible for the governance of the study with reference number 11/CMC/5299. The Cardiff and Vale University Health Board Research & Development Office approved the proposal in 2013. Written informed consent was received from each participant with a different consent and patient information sheet (PIS) for healthy controls. All PIS and consent forms were approved by the ethics committee.

## Author Contributions

CT: study design, data collection, and analysis and writing of article. RD: study design and data collection. AW, GJ, and EC: study design, analysis, and writing of article.

## Conflict of Interest

The authors declare that the research was conducted in the absence of any commercial or financial relationships that could be construed as a potential conflict of interest.

## Abbreviations

APC: antigen presenting cells
MHC: major histocompatibility complex
HLA: human leucocyte antigen
CD: cluster of differentiation
TNF: tumor necrosis factor
RA: rheumatoid arthritis
Treg: regulatory T cell
CMV: cytomegalovirus
NK: natural killer cells
UHW: university hospital of wales
ACR: American college of rheumatology
DAS28: disease activity score 28
RF: rheumatoid factor
ACPA: anti-citrullinated peptide antigen
ESR: erythrocyte sedimentation ratio
CRP: C reactive protein
PBMCs: peripheral blood mononuclear cells.
